# Subtype distribution of lymphomas in northwestern Iran: a retrospective analysis of 659 cases according to World Health Organization classification

**DOI:** 10.1186/s12885-022-10132-2

**Published:** 2022-10-12

**Authors:** Javad Jalili, Amir Vahedi, Amin Danandehmehr, Parya Aynechi, Ali Esfahani, Yousef Roosta, Hooman Nateghian, Amirhosein Ghafouri Asbagh, Fateme Hajihoseinlou

**Affiliations:** 1 Radiology Department, Faculty of Medicine, Tabriz University of Medical Sciences, Tabriz, Iran; 2 Department of Pathology, Tabriz University of Medical Sciences, Tabriz, Iran; 3 Department of Internal Medicine, School of Medicine and Allied Sciences, Ardabil University of Medical Sciences, Ardabil, Iran; 4 Hematology and Oncology Research Center, Tabriz University of Medical Sciences, Tabriz, Iran; 5 Student Research Committee, Tabriz University of Medical Sciences, Tabriz, Iran; 6 Internal Medicine Department, Urmia University of Medical Sciences, Urmia, Iran; 7 Research Center for Evidence‑Based Medicine, Iranian EBM Centre: A Joanna Briggs Institute Affiliated Group, Tabriz University of Medical Sciences, Tabriz, Iran

**Keywords:** Lymphoma, Non-hodgkin lymphoma, Lymphoma subtype, Incidence, Iran

## Abstract

**Background:**

The distribution of lymphoma subtypes differs strikingly by geographic variations. However, there is limited information on this research in northern Iran. This study aims to evaluate the incidence, subtype, age, sex, and extranodal distribution of lymphomas diagnosed according to the latest WHO classification in a large referral center in northwest Iran.

**Methods:**

In a retrospective study, the medical records of all patients with a diagnosis of lymphoma made between 2018 and 2021 were retrieved from the pathology archive of Imam Reza Medical Center, Tabriz. Lymphoma diagnosis was also made based on the appreciation of morphologic and immunophenotypic features and genetic characteristics in the context of clinical presentation.

**Results:**

This study includes a total of 659 patients with lymphoma diagnosed from 2018 to 2021. The number of lymphoma patients were increased each year, with 51 (7.7%), 96 (14.6%), 244 (40.7%), and 268 (40.7%) reported from 2018 to 2021, respectively. 59% of the patients were men. The participants’ mean age was 50.5 ± 19.8 years, while the mean age at diagnosis was 49.3 ± 19.6 years. 2.1% were precursor lymphoid neoplasm, 61.6% were mature B cell neoplasm, 8.8% were mature T cell neoplasm, and 27.5% were Hodgkin lymphoma. The most prevalent subtype of mature B-cell lymphoma was DLBCL (55.1%), followed by SLL (18.7%). Extranodal involvement was seen in 40.5% of all cases.

**Conclusion:**

The subtype distribution of lymphomas in northwest Iran is reported and compared with studies all over the world and inside Iran.

## Introduction

Lymphoma is a diverse category of hematological malignancies originating from lymphocytes, with a wide range of underlying etiological factors, clinical presentations, histological features, molecular profiles, and treatment options [1]. The incidence of lymphoma also varies geographically, with the greatest rates in North America and Western Europe and much lower rates in East Asia. However, the total incidence of lymphoma is growing in Asian countries [2]. The Middle East and Iran have a greater prevalence of lymphomas than Western countries [3]. Non-Hodgkin lymphoma (NHL) accounts for 3 to 7% of all cancers in various areas of Iran, ranking fifth and eighth in men and women, respectively [4].

Lymphoma is classified into Hodgkin’s lymphoma (HL) and non-Hodgkin’s lymphoma (NHL). NHL accounts for approximately 90% of lymphoid neoplasms worldwide and is divided into precursor lymphoid neoplasms, mature B cell neoplasms, and mature T/natural killer (NK)-cell neoplasms. Each classification can be further subdivided based on cell differentiation stage, histological appearance, clinical characteristics, immunological phenotypes, and molecular and cytogenetic variations [5, 6]. More than 80 mature lymphoma entities are recognized in the World Health Organization classification of lymphoid neoplasms, which was amended in 2017. Following the principles of lymphoma classification, lymphoma [5, 7], diagnosis is also based on the appreciation of morphologic and immunophenotypic features and genetic characteristics in the context of clinical presentation [5, 7].

Over the last two decades, substantial studies have suggested environmental impacts and significant etiologic diversity among NHL subtypes[8]. Although genetic and ethnic differences, as well as environmental variables such as socioeconomic issues, have been proposed to explain these geographical differences in the incidence rates and distribution of subtypes, the cause of these differences remains largely unexplained [9]. Comparing incidence, subtypes, and other clinical data in different parts of the world may provide new clues for future etiologic investigations and treatments. This study aims to evaluate the incidence, subtype, age, sex, and nodal/extranodal distribution of lymphomas diagnosed according to the latest WHO classification in a large referral center in Northwest Iran from 2018 to 2021.

### Methods

In a retrospective study, the medical records of all patients with a diagnosis of lymphoma made between 2018 and 2021 were retrieved from the pathology archive of Imam Reza Medical Center, Tabriz. This center is affiliated with Tabriz University of Medical Sciences, one of the reference centers in the northwestern region of Iran. Patient information, including demographics (age, gender), initial site involvement (nodal or extranodal), date and age at the diagnosis were collected from the submission forms of pathological sampling and medical registering. Inclusion criteria included all lymphoma cases diagnosed by a specialist physician and classified according to the latest WHO classification of Tumors of Haematopoietic and Lymphoid Tissue. Exclusion criteria included lymphatic leukemias involving the brain and peripheral blood, plasma cell neoplasm, and immunodeficiency-related lymphatic disorders. Cases with inadequate information or inconclusive histopathological findings were also excluded. The primary diagnosis was made based on formalin-fixed paraffin sections of three-micron thick slides stained with Hematoxylin and Eosin (H&E), Periodic Acid Schiff (PAS), Giemsa, silver, and cholera acetate esterase. In cases in which histochemical methods did not yield conclusive results, the diagnosis and the differentiation of Hodgkin’s lymphoma and non-Hodgkin’s lymphoma, from epithelial, mesenchymal, and myeloproliferative lesions, were made using immunohistochemical staining of paraffin-embedded sections methods with the following markers: CD3, CD4, CD5, CD8, CD10, CD15, CD20 (L26), CD21, CD23, CD30, CD43, CD45(LCA), CD56, CD79, CD99, Anaplastic large cell lymphoma kinase-1(ALK-1), Epithelial Membrane Antigen(EMA), cyclin D1, BCL-2, BCL-6, MuM1, Kappa(K), Lambda (λ), LMP, Ki-67, EBER, TdT. Flowcytometry (on lymph node aspirates or tissue chops), karyotype on fresh tissue (when available), polymerase chain reaction (PCR) for detection of clonality of IgH and/or TCR gene rearrangement, and in situ hybridization for detection of EBV-encoded small RNA (EBER) and diagnostic translocations were performed. The latter was sometimes beneficial in confirming follicular and Burkitt lymphoma and differentiating other high-grade B cell lymphomas from the latter. IBM SPSS Statistics for Windows, version 26 (IBM Corp., Armonk, N.Y., USA) was used for all statistical analyses. We applied the terms of mean (± standard deviation) to describe continuous variables and count (frequency) to describe other nominal variables. All data were obtained with the approval of the ethics committee of Tabriz University of Medical Sciences.

## Results

**Fig. 1:**
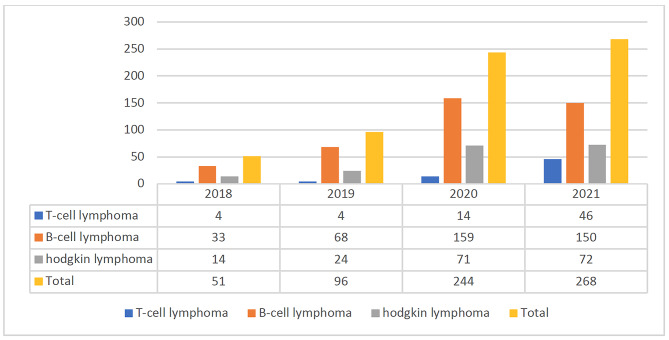
frequency of different types of lymphomas in Northern Iran from 2018 to 2021

This study includes a total of 659 patients with lymphoma diagnosed from 2018 to 2021. During the study, the number of lymphoma patients were increased each year, with 51 (7.7%), 96 (14.6%), 244 (40.7%), and 268 (40.7%) reported from 2018 to 2021, respectively. As shown in Fig. 1, B-cell lymphoma had the highest incidence rate among all patients diagnosed each year, whereas T-cell lymphoma had the lowest number of patients diagnosed over the study years.

In the current study, 59% of the patients were men. The participants’ mean age was 50.5 ± 19.8 years, while the mean age at diagnosis was 49.3 ± 19.6 years. Extranodal lymphoma is defined as lymphoma that arises outside of the lymph nodes and thymus. When found in bone marrow, it was confined and lacked lymphoma cell periphery. The mediastinum was also considered to be an extranodal site. In our research, around 40% of the patients showed extranodal involvement. Fourteen cases (2.1% of all) were precursor lymphoid neoplasm, 406 cases (61.6% of all) were mature B cell neoplasm, 58 cases (8.8% of all) were mature T cell neoplasm, and 181 cases (27.5% of all) were Hodgkin lymphoma. Four patients (28.5%) of the 14 precursor lymphoid neoplasms were B-lymphoblastic lymphoma, and 10 cases (71.4%) were T-lymphoblastic lymphoma. Of the 181 patients diagnosed with Hodgkin lymphoma, 156 (86.1%) were classical type (CHL), and the rest (13.8%) were nodular lymphocyte predominant Hodgkin lymphoma (NLPHL) (Table 1). The most prevalent subtype of mature B-cell lymphoma (55.1%) was diffuse large B cell lymphoma (DLBCL), followed by small lymphocytic lymphoma (SLL) (18.7%), MALTOMA (9.1%), follicular lymphoma (FL) (4.9%), and both mantle cell lymphoma (MCL) and Burkitt lymphoma (BL) (3.2% ). There were also other less common subtypes, the details of which are provided in Table 1. Among the 58 cases with a diagnosis of mature T-cell neoplasm, the most prevalent subtype was peripheral T cell lymphoma (PTCL) (44.8%), followed by LP (39.6%), anaplastic large cell lymphoma, ALK-negative (ALCL, ALk negative) (5.1%), hepatosplenic T-cell lymphoma (HSTL) and mycosis fungoides (MF) and angioimmunoblastic T cell lymphoma (AITCL), all with the 3.4% incidence rate among the T-cell neoplasms. The male/female ratio was 1.4 in total, 2.5 for immature lymphoid neoplasms, 1.4 for mature B cell neoplasms, 2.0 for mature T cell neoplasms, and 1.2 for Hodgkin lymphomas (Table 1). The highest M/F ratio was seen in PTCL (4.2), BL (3.3), and DLBCL (2) , respectively.

**Table 1:** Distribution of lymphomas and pertaining demographic factors in Northern Iran according to WHO classification

Type	Subtype	NO.	Male	Female	M/F	Mean age	Mean age (Diagnosis)	Nodal	Extranodal	N/E
Total		659 (100)	389 (59)	270 (41)	1.4	50.5 ± 19.8	49.3 ± 19.6	392 (59.5)	267 (40.5)	1.4
Precursor lymphoma	B-LBL	4 (0.6)	4	0	-	48 ± 29.6	33 ± 23.5	2	2	1
	T-LBL	10 (1.5)	6	4	1.5	25.1 ± 23.6	24.7 ± 23.6	9	3	3
	Total	14 (2.1)	10	4	2.5	28.91 ± 24.85	27.07 ± 23.06	11	5	2.2
Hodgkin lymphoma	CHL	156 (23.7)	89	67	1.3	38.7 ± 19.6	36.5 ± 18.2	127	29	4.3
	NLPHL	25 (3.8)	11	14	0.7	41.5 ± 20.2	39.3 ± 18	23	2	11.5
	Total	181 (27.4)	100	81	1.2	39.13 ± 19.67	36.92 ± 18.22	150	31	4.8
B Cell lymphoma	SLL (CLL)	76 (11.5)	44	32	1.3	59.5 ± 12.6	57.8 ± 14.4	27	49	0.5
	MCL	13 (2)	7	6	1.1	53.9 ± 14.1	56.7 ± 13.9	6	7	0.8
	FL	20 (3)	8	12	0.6	50.9 ± 11.1	50.5 ± 10.1	9	9	1
	LPL	1 (0.2)	0	1	-	46	45	1	0	-
	MALTOMA	37 (5.6)	19	18	1.05	64.2 ± 14.7	61.7 ± 14.9	17	20	0.8
	DLBCL	224 (34)	137	87	1.5	55.9 ± 16.6	55.7 ± 16.9	125	101	1.2
	TCHLBL	3 (0.5)	3	0	-	36.3 ± 17.8	36 ± 17.3	1	2	0.5
	GZ	11 (1.7)	6	5	1.2	55.7 ± 17.1	55.8 ± 15.5	13	0	-
	DLBCL (Leg)	3 (0.5)	2	1	2	50 ± 28.1	48 ± 27.2	0	3	-
	EBV positive large B cell	1 (0.2)	0	1	-	50	50	1	0	-
	DLBCL (Mediastinum)	2 (0.3)	2	0	-	52 ± 33.9	50.5 ± 34.6	0	2	-
	PBL	2 (0.3)	2	0	-	53	40 ± 16.9	0	2	-
	BL	13 (2)	10	3	3.3	23.2 ± 20.7	22.2 ± 20.8	11	4	2.7
	Total	406 (61.6)	240	166	1.4	55.63 ± 17.37	54.71 ± 17.54	202	199	1.01
T Cell lymphoma	HSTL	2 (0.3)	0	2	-	30	30	0	2	-
	MF	2 (0.3)	2	0	-	38	38	0	2	-
	LP	23 (3.5)	11	12	0.9	65.4 ± 11.3	62.8 ± 12.9	7	19	0.3
	PTCL	26 (3.9)	21	5	4.2	52.7 ± 13.9	54.1 ± 15.5	17	9	1.8
	AITCL	2 (0.3)	2	0	-	20 ± 0	20 ± 0	2	0	-
	ALCL (ALK-negative)	3 (0.5)	3	0	-	42 ± 14.1	37.3 ± 11.8	3	0	-
	Total	58 (8.8)	39	19	2.0	56.27 ± 18.14	54.30 ± 16.98	29	32	0.9

B-LBL: B-lymphoblastic lymphoma, T-LBL: T-lymphoblastic lymphoma, CHL: Classical Hodgkin lymphoma, NLPHL: nodular lymphocyte predominant Hodgkin lymphoma, DLBCL: Diffuse large B cell lymphoma, SLL: Small lymphocytic lymphoma, FL: Follicular lymphoma, MCL: Mantle cell lymphoma, BL: Burkitt lymphoma, PTCL: Peripheral T cell lymphoma, ALCL: Anaplastic large cell lymphoma, MF: mycosis fungoides, HSTL: hepatosplenic T-cell lymphoma, AITCL: angioimmunoblastic T cell lymphoma, LP: lymphomatoid papulosis, PBL: plasmablastic lymphoma, GZ: gray zone lymphoma, TCHLBL: T cell histiocyte large B cell lymphoma

Extranodal involvement was 1.8% in precursor lymphoid neoplasms, 74.5% in mature B cell neoplasms, 11.9% in mature T cell neoplasms, and 11.6% in Hodgkin lymphomas. DLBCL, SLL, and MALTOMA were the three most common kinds of mature B cell neoplasm with extranodal involvement, in order of frequency (Tables 1 and 2). LP among mature T cells, and CHL among Hodgkin lymphoma, were the subtypes with the most extranodal involvement. Overall, the three common sites of extranodal involvement were bone marrow (46.8% of extranodal involvement), thoracic region (12.3%), and soft tissue (9.3%) (Table 2). The other sites of extranodal involvement are listed in Table 2.

**Table 2:** Distribution of extranodal sites of lymphomas

Subtypes	SLL (CLL)	DLBCL	FL	LP	TLBL	MALTOMA	CHL	BL	PTCL	Others	Total (%)
**Sites**											
Bone	2	9				1	1			2	15 (5.6)
Nasal and oral cavity and tonsils	2	5				1	2				10 (3.7)
Thyroid		1				1					2 (0.7)
Thoracic region	2	13		2	1		9		2	4	33 (12.3)
Soft tissue		19	1			3	1	1			25 (9.3)
Testis		1									1 (0.3)
GI		5					1	1			7 (2.6)
Spleen		3		1			1			3	8 (2.9)
CNS		4									4 (1.4)
Skin									1	3	4 (1.4)
BM	34	31	8	16	2	11	7	2	3	11	125 (46.8)
Retro peritoneum	2	2									4 (1.4)
Pancreas		1									1 (0.3)
Ovary	1										1 (0.3)
Peripheral blood	4						4				8 (2.9)
Orbit						2					2 (0.7)
Lung		4					3		3	1	11 (4.1)
Liver						1					1 (0.3)
Kidney	2	3									5 (1.8)
Total (%)	49 (18.3)	101 (37.8)	9 (3.3)	19 (7.1)	3 (1.1)	20 (7.4)	29 (10.8)	4 (1.4)	9 (3.3)	24 (8.9)	267

GI: gastrointestinal, CNS: central nervous system, BM: bone marrow

**Fig. 2:**
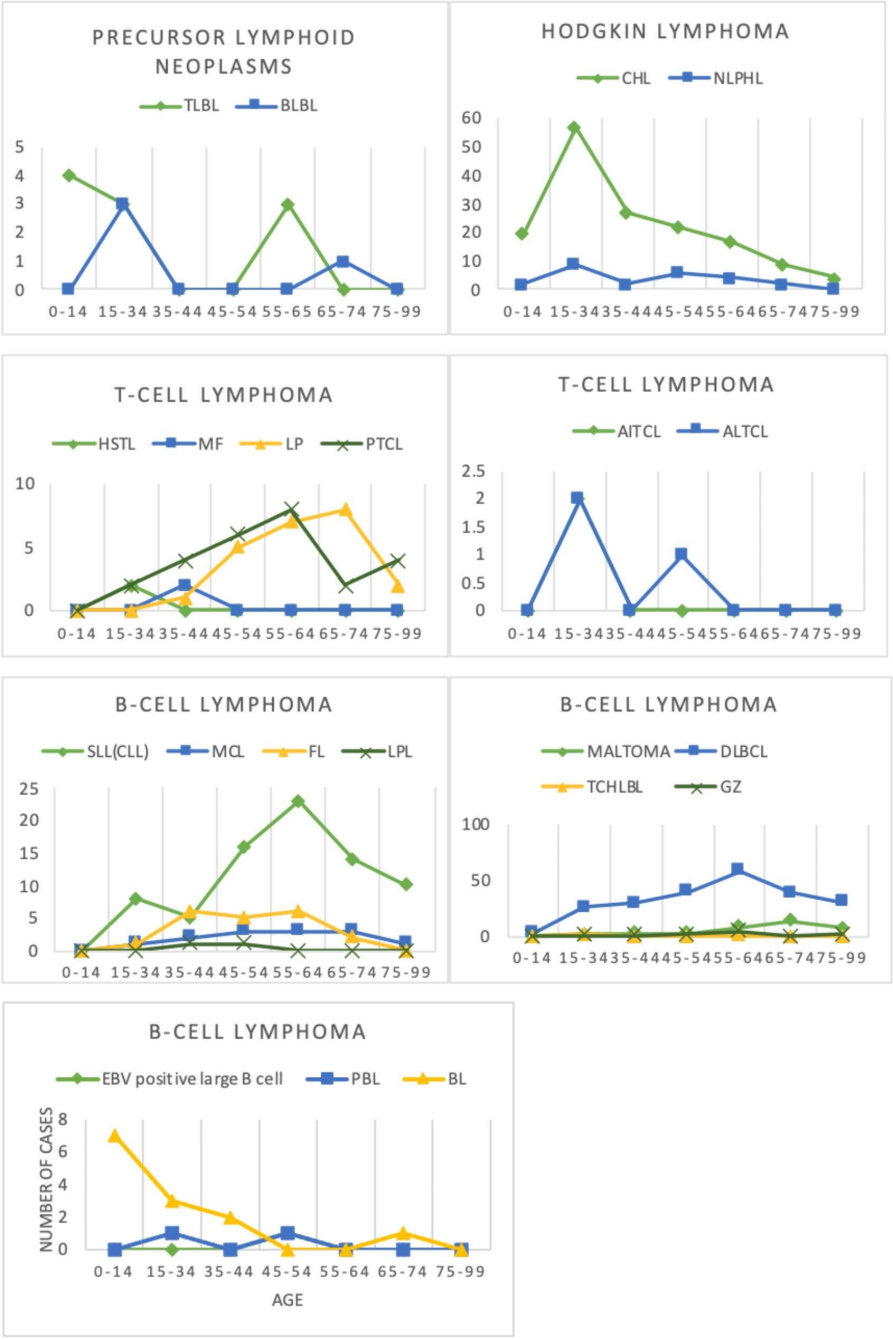
Age-specific distribution of diagnosed lymphomas

The age-specific incidence rate of lymphomas is demonstrated in Fig. 2. Patients with precursor lymphoid neoplasms had an average age of 28.91 ± 24.85 years. The fact that this kind of lymphoma was more common at younger ages was noteworthy. TLBL, in particular, had the greatest frequency among children aged 0 to 14 years. The average age of Hodgkin lymphoma patients was 36.92 ± 18.22 years. CHL had a significantly higher incidence rate among these lymphomas in the age range of 15–34 years. The majority of mature B cell lymphomas were seen in adults with a mean age of 55.63 ± 17.37. However, the prevalence of BL is greatest in children and decreases in adulthood. The average age of T cell lymphoma patients was 56.27 ± 18.14 years, and the majority of this form of lymphoma was found in adults, with the largest incidence of LP and PTCL occurring between the ages of 65–74 and 55–64 years, respectively.

## Discussion

Epidemiologic studies suggest that the distribution of lymphoma subtypes differs strikingly by geographic variations. However, there is limited information on this research in Northern Iran. This report demonstrates the most extensive descriptive study of subtype distribution of lymphomas classified by the WHO criteria in a single institution in Iran.

Recent studies have reported HL frequency among other types of lymphoma to be less than 10% in various regions [10– 12]. The latest research on cancer statistics in US HL made up 9.59% of all lymphomas [13]. In our observation, this figure was 27.5%, with all cases being CHL except for two that were NLPHL. With other studies in Iran reporting higher than worldwide average HL frequency among other types of lymphomas (10 ~ 40%) [3, 4, 14, 15], it can be said that Iran has one of the highest HL proportions among other lymphomas in the world. Other countries in the regions of Middle East and North Africa, such as Jordan, Iraq, Bahrain, and Lebanon have also reported high percentages of HL, ranging from 24 to 39%, indicating a similar HL distribution pattern [16– 19]. This difference can be due to Iran’s population structure, as children and young adults comprise the majority of Iran’s population. In Western countries, HL shows a bimodal age distribution with a peak incidence in the third and sixth decades of life. In contrast, a high incidence in childhood is seen in developing countries. In addition, HL shows a gradual decline in cases with age in this series, consistent with what was seen in China [15, 16, 20, 21].

BCL compromised 61.6% of all lymphomas reported in this study, similar to those reported in MENA countries [17, 18, 22]. However, a greater number was reported in most East Asian countries such as South Korea (72.5%), China (64.4%), Sri Lanka (65.1%), and Thailand (78.3%) [23– 26]. BCL constitutes a large portion of NHL in US (83.5%) and Europe (79.9%) as well [27, 28].

In accordance with the literature, DLBCL is the most common subtype of B-cell lymphomas in Iran, with SLL being the second in this report. However, BL was the second most common subtype in another study investigating southern Iran [4].

Previous studies reported FL as the second most common NHL subtype with slightly varying percentages [29]. However, in several newer studies, SLL/CLL has been reported as the second most common NHL subtype. Al-Hamadani et al. examined the data of about half a million NHL cases registered in the US from 1998 to 2011, and DLBCL (32.5%), CLL/SLL (18.6%), and FL (17.1%) were the three most common subtypes in US [27].

Geographical variation in FL distribution has long been recognized. This difference in follicular lymphoma incidence is attributed to different molecular pathways that lead to lymphoma in patients from different regions. There is also the possibility that some cases were diagnosed only after transformation to diffuse large B-cell lymphoma due to a lack of significant symptoms in low-grade lymphomas. This was inferred by the significantly lower incidence of bcl2 gene rearrangements, the hallmark of FL, in Asian compared to western countries. Naresh et al. proposed that the lower rates of FL compared to DLBCL in developing countries may be due to many DLBCLs having progressed from previously undiagnosed FLs, besides the unique regional, genetic, or environmental factors that might have contributed to such progression [18, 19, 30].

One known major risk factor for mature BCL is immune system abnormality. Immune deficient patients have a markedly increased risk of BCL, particularly DLBCL and BL. Some autoimmune diseases such as Sjogren’s syndrome or Hashimoto’s thyroiditis are also associated with an increased risk of developing BCLs [31, 32].

It is commonly thought that mature T cell neoplasms display higher rates in the Asian continent than in others. Therefore, racial predisposition has been proposed as a risk factor for T/NK lymphomas [33].

The current study and similar reports from some of the surrounding Middle East countries, as well as the western countries, show a low relative proportion of T-cell lymphomas. This is in contrast to its high proportion in East Asian countries like Japan, China, and Korea, constituting 27 − 30.5% of NHLs. While in our population, T/NK lymphomas were seen in under 10% of patients. Therefore, racial predisposition has been proposed as a risk factor for T/NK lymphomas [34, 35]. Such significant variation in the geographical distribution of T-cell lymphomas has long been recognized and has been attributed to racial predisposition, HTLV-I viral infection, and lower relative incidence of B-cell NHL in the Far East [15].

A previous large multicenter retrospective study confirmed the geographic variations and reported the high frequency of ATLL in Japan, AITL and ETCL in Europe, ALCL and ALK-positive in North America, and ENKTCL in Asian countries other than Japan [36].

As for age-specific incidence, two peaks were seen in precursor lymphoma and TCL age distribution; however, the reported frequency of these subtypes are very low, and no robust generalization can be made. No other bimodal distribution was found for other types of lymphomas.

HL exhibits a gradual decrease in cases with age in this study, consistent with what was seen in China. In contrast, a bimodal age curve (a mode at 15 ~ 35 years of age and the second mode in elderly) is apparent in North America [21, 24].

In our study, similar to other reports, a male preponderance was seen in most lymphoma subtypes [24, 34, 37]. This difference was more prominent in TCL and precursor lymphoma cases.

In this study, Extranodal involvements were seen in 20.6% of HL cases and 49.3% of NHL cases. Different countries of the world have reported varying frequencies of extranodal lymphoma cases, for example, India (22%), Iraq (48.3%), China (53.5%), and Korea (53.5%) [18, 24, 38, 39]. Diverse definition criteria, ethnic and genetic factors may cause the fluctuating frequency of extranodal lymphomas.

As reported in previous studies, DLBCL is the most common extranodal lymphoma, which is consistent with our results (37.8%) [38, 40]. Additionally, in this study, extranodal NHL most commonly involved bone marrow, whereas the GI tract, Waldeyer’s ring, nose, and sinuses are the common sites in the literature [41, 42]. Geographical differences in Helicobacter pylori incidence also may play a key role in this difference. The fluctuating frequency of extranodal lymphomas may also be caused by genetic and ethnic factors and diverse definition criteria [35].

In nodal involvement, some lymph node sites have shown a disproportionate prevalence of specific lymphoma subtypes. Laurent et al. examined 938 lymphoma cases in France and the most frequent sites were cervical lymph nodes (36.8% of all cases), inguinal lymph nodes (16.4%), axillary lymph nodes (11.9%), and supraclavicular lymph nodes (11%) [43].

The frequency of lymphomas in Iran is rising, and the increasing trend seen in previous studies in Iran and other countries was also seen in our study [44]. Global patterns of non-Hodgkin lymphoma were studied by Miranda et al., and it was reported that most populations exprienced stable or barely increasing incidence rates [45]. Singh et al. predicted a 30% increase in HL cases for 2040 reaching 107,000 cases [35].

Considering that the lymphoma cases, including consultation cases, were referred from all North West regions of Iran, the findings may represent the distribution of lymphoma subtypes in Northern Iran.

## Conclusion

In conclusion, in the current study, the subtype distribution of lymphomas in northern Iran is reported and compared with studies all over the world and inside Iran. HL has a greater share of lymphomas reported in Iran compared to countries outside the MENA region, with the majority of the cases being CHL. A high percentage of extranodal lymphomas are reported, including a relatively high frequency of bone marrow involvement. This disease’s occurrence differs geographically, showing that regional factors may play an essential role in its etiology. Additional research should be done to recognize the effects of these factors on the incidence of lymphomas.

## Data Availability

(ADM) The datasets generated during and/or analyzed during the current study are available at https://data.mendeley.com/datasets/wg28r74x42.

## References

[1] Matasar MJ, Zelenetz AD. Overview of lymphoma diagnosis and management. Radiol Clin North Am. 2008 Mar;46(2):175–98. 10.1016/j.rcl.2008.03.005 18619375

[2] Armitage JO, Gascoyne RD, Lunning MA, Cavalli F (2017). Non-Hodgkin lymphoma. Lancet (London England).

[3] Hashemi-Bahremani M, Parwaresch MR, Tabrizchi H, Gupta RK, Raffii MR (2007). Lymphomas in Iran. Arch Iran Med.

[4] Monabati A, Safaei A, Noori S, Mokhtari M, Vahedi A (2016). Subtype distribution of lymphomas in South of Iran, analysis of 1085 cases based on World Health Organization classification. Ann Hematol.

[5] Swerdlow SH, Campo E, Harris NL, Jaffe ES, Pileri SA, Stein HTJ. WHO Classification of Tumours of Haematopoietic and Lymphoid Tissues. WHO; 2017. 586 p.

[6] Swerdlow SH, Campo E, Pileri SA, Lee Harris N, Stein H, Siebert R (2016). The 2016 revision of the World Health Organization classification of lymphoid neoplasms. Blood.

[7] De Leval L, Jaffe ES (2020). Lymphoma Classification. Cancer J.

[8] Chiu BCH, Hou N. Epidemiology and etiology of non-hodgkin lymphoma. Cancer Treat Res. 2015;165. 10.1007/978-3-319-13150-4_1 25655604

[9] Müller AMS, Ihorst G, Mertelsmann R, Engelhardt M (2005). Epidemiology of non-Hodgkin’s lymphoma (NHL): trends, geographic distribution, and etiology. Ann Hematol.

[10] Perry AM, Diebold J, Nathwani BN, Maclennan KA, Müller-Hermelink HK, Bast M (2016). Non-Hodgkin lymphoma in the developing world: review of 4539 cases from the International Non-Hodgkin Lymphoma Classification Project. Haematologica.

[11] Perry AM, Diebold J, Nathwani BN, Maclennan KA, Müller-Hermelink HK, Bast M (2016). Relative frequency of non-Hodgkin lymphoma subtypes in selected centres in North Africa, the middle east and India: A review of 971 cases. Br J Haematol.

[12] Cao C, Feng J, Gu H, Tang H, Xu L, Dong H (2018). Distribution of lymphoid neoplasms in Northwest China: Analysis of 3244 cases according to WHO classification in a single institution. Ann Diagn Pathol.

[13] Siegel RL, Miller KD, Fuchs HE, Jemal A (2022). Cancer statistics, 2022. CA Cancer J Clin.

[14] Basirat M, Rabiei M, Bashardoustand N (2016). Incidence of head and neck Lymphoma in Guilan Province, Iran. Asian Pac J Cancer Prev.

[15] Samiee F, Mohammadi R, Shirian S, Alijani MR, Aledavood A, Negahban S (2021). Spectrum of lymphoma subtypes based on the latest World Health Organization classification in southern Iran from 2000 to 2011. Future Oncol.

[16] Aladily TN, Khreisat W, Ashukhaibi O, Alkhatib SM, Annab H, Tarawneh MS (2021). The epidemiology of lymphoma in Jordan: A nationwide population study of 4189 cases according to World Health Organization classification system. Hematol Oncol Stem Cell Ther.

[17] Aljufairi EA, George SM, Alshaikh SA, Radhi AA, Mohamed RM (2018). Spectrum of lymphoma in Bahrain. A retrospective analysis according to the World Health Organization classification. Saudi Med J.

[18] Yaqo RT, Hughson MD, Sulayvani FK, Al-Allawi NA (2011). Malignant lymphoma in northern Iraq: A retrospective analysis of 270 cases according to the World Health Organization classification. Indian J Cancer.

[19] Sader-Ghorra C, Rassy M, Naderi S, Kourie HR, Kattan J (2014). Type distribution of lymphomas in Lebanon: five-year single institution experience. Asian Pac J Cancer Prev.

[20] Yang I-H (2014). Neurovascular Injury in Hip Arthroplasty. Hip Pelvis.

[21] Nakatsuka SI, Aozasa K (2006). Epidemiology and pathologic features of Hodgkin lymphoma. Int J Hematol.

[22] Touma E, Antoun L, Hallit S, Nasr F, Massoud M, El Othman R, et al. Non Hodgkin lymphoma in Lebanon: a retrospective epidemiological study between 1984 and 2019. BMC Public Health. 2021 Dec;21(1). 10.1186/s12889-021-11840-3 PMC8501727 34627178

[23] Yoon SO, Suh C, Lee DH, Chi HS, Park CJ, Jang SS (2010). Distribution of lymphoid neoplasms in the Republic of Korea: analysis of 5318 cases according to the World Health Organization classification. Am J Hematol.

[24] Yang QP, Zhang WY, Yu JB, Zhao S, Xu H, Wang WY, et al. Subtype distribution of lymphomas in Southwest China: analysis of 6,382 cases using WHO classification in a single institution. Diagn Pathol. 2011 Aug;6(1). 10.1186/1746-1596-6-77 PMC3179701 21854649

[25] Intragumtornchai T, Bunworasate U, Wudhikarn K, Lekhakula A, Julamanee J, Chansung K (2018). Non-Hodgkin lymphoma in South East Asia: An analysis of the histopathology, clinical features, and survival from Thailand. Hematol Oncol.

[26] Waravita TS, Wijetunge TS, Ratnatunga NVI (2015). Pattern of lymphoma subtypes in a cohort of Sri Lankan patients. Ceylon Med J.

[27] Al-Hamadani M, Habermann TM, Cerhan JR, Macon WR, Maurer MJ, Go RS (2015). Non-Hodgkin lymphoma subtype distribution, geodemographic patterns, and survival in the US: A longitudinal analysis of the National Cancer Data Base from 1998 to 2011. Am J Hematol.

[28] Costas L, Casabonne D, Benavente Y, Becker N, Boffetta P, Brennan P (2012). Reproductive factors and lymphoid neoplasms in Europe: findings from the EpiLymph case-control study. Cancer Causes Control.

[29] Mozaheb Z, Aledavood A, Farzad F (2011). Distributions of major sub-types of lymphoid malignancies among adults in Mashhad, Iran. Cancer Epidemiol.

[30] Naresh KN, Srinivas V, Soman CS. Distribution of various subtypes of non-Hodgkin’s lymphoma in India: A study of 2773 lymphomas using R.E.A.L. and WHO classifications. Ann Oncol. 2000;11(SUPPL. 1). 10707782

[31] Fragkioudaki S, Mavragani CP, Moutsopoulos HM. Predicting the risk for lymphoma development in Sjogren syndrome: An easy tool for clinical use. Medicine (Baltimore). 2016 Jun;95(25). 10.1097/MD.0000000000003766 PMC4998301 27336863

[32] Chen YK, Lin CL, Cheng FTF, Sung FC, Kao CH (2013). Cancer risk in patients with Hashimoto’s thyroiditis: a nationwide cohort study. Br J Cancer.

[33] Ren W, Li W, Ye X, Liu H, Pan-Hammarström Q (2017). Distinct subtype distribution and somatic mutation spectrum of lymphomas in East Asia. Curr Opin Hematol.

[34] Nair R, Arora N, Mallath MK (2016). Epidemiology of Non-Hodgkin’s Lymphoma in India. Oncol.

[35] N DPS, E S-F DSSVAG. F B. Global patterns of Hodgkin lymphoma incidence and mortality in 2020 and a prediction of the future burden in 2040. Int J cancer. 2022;150(12). 10.1002/ijc.33948 35080783

[36] Vose JM, Neumann M, Harris ME (2008). International peripheral T-cell and natural killer/T-cell lymphoma study: pathology findings and clinical outcomes. J Clin Oncol.

[37] Bray F, Ferlay J, Soerjomataram I, Siegel RL, Torre LA, Jemal A (2018). Global cancer statistics 2018: GLOBOCAN estimates of incidence and mortality worldwide for 36 cancers in 185 countries. CA Cancer J Clin.

[38] Padhi S, Paul TR, Challa S, Prayaga AK, Rajappa S, Raghunadharao D (2012). Primary extra nodal non Hodgkin lymphoma: a 5 year retrospective analysis. Asian Pac J Cancer Prev.

[39] Sim J, Takayama T, Cho J, Kim SJ, Kim WS, Ree HJ, et al. Changing trends in lymphoid neoplasm distribution in South Korea: analysis of 8615 cases from a single institute, 1997–2016: An observational study. Medicine (Baltimore). 2019 Nov;98(45):e17641–e17641. 10.1097/MD.0000000000017641 PMC6855639 31702615

[40] Das J, Ray S, Sen S, Chandy M (2014). Extranodal involvement in lymphoma – A Pictorial Essay and Retrospective Analysis of 281 PET/CT studies. Asia Ocean J Nucl Med Biol.

[41] Franco Cavalli Emanuele Zucca HS. EXTRANODAL LYMPHOMAS: pathology and management. CRC PRESS; 2019.

[42] Ferry JA (2008). Extranodal lymphoma. Arch Pathol Lab Med.

[43] Laurent C, Do C, Gourraud PA, De Paiva GR, Valmary S, Brousset P. Prevalence of Common Non-Hodgkin Lymphomas and Subtypes of Hodgkin Lymphoma by Nodal Site of Involvement: A Systematic Retrospective Review of 938 Cases. Medicine (Baltimore). 2015 Jun;94(25):e987–e987. 10.1097/MD.0000000000000987 PMC4504656 26107683

[44] Akbari ME, Bastani Z, Mokhtari S, Atarbashi Moghadam S (2015). Oral Lymphoma Prevalence in Iranian Population: A Multicenter Retrospective Study. Iran J cancer Prev.

[45] Miranda-Filho A, Piñeros M, Znaor A, Marcos-Gragera R, Steliarova-Foucher E, Bray F. Global patterns and trends in the incidence of non-Hodgkin lymphoma. Cancer Causes Control. 2019;30(5). 10.1007/s10552-019-01155-5 30895415

